# The HACMS program: using formal methods to eliminate exploitable bugs

**DOI:** 10.1098/rsta.2015.0401

**Published:** 2017-09-04

**Authors:** Kathleen Fisher, John Launchbury, Raymond Richards

**Affiliations:** 1 Department of Computer Science, Tufts University, Medford, MA, USA; 2 DARPA, Arlington, VA, USA

**Keywords:** formal methods, high-assurance software, cybersecurity

## Abstract

For decades, formal methods have offered the promise of verified software that does not have exploitable bugs. Until recently, however, it has not been possible to verify software of sufficient complexity to be useful. Recently, that situation has changed. SeL4 is an open-source operating system microkernel efficient enough to be used in a wide range of practical applications. Its designers proved it to be fully functionally correct, ensuring the absence of buffer overflows, null pointer exceptions, use-after-free errors, etc., and guaranteeing integrity and confidentiality. The CompCert Verifying C Compiler maps source C programs to provably equivalent assembly language, ensuring the absence of exploitable bugs in the compiler. A number of factors have enabled this revolution, including faster processors, increased automation, more extensive infrastructure, specialized logics and the decision to co-develop code and correctness proofs rather than verify existing artefacts. In this paper, we explore the promise and limitations of current formal-methods techniques. We discuss these issues in the context of DARPA’s HACMS program, which had as its goal the creation of high-assurance software for vehicles, including quadcopters, helicopters and automobiles.

This article is part of the themed issue ‘Verified trustworthy software systems’.

## 1. Pervasive cybersecurity threats

To a first approximation all computers are networked. Even many systems that are supposedly air-gapped are periodically connected, often via USB keys, so their software can be updated. This connectivity greatly increases the usefulness of such systems, but it also introduces the risk of remote hacking.

The security vulnerabilities of laptop and desktop computers have led to the pervasive use of anti-virus and intrusion-detection software to mitigate the risks. The emerging Internet of Things means that we also have to worry about the security of many seemingly mundane objects: everything from SCADA systems that control industrial infrastructure like sewage treatment plants and prison doors, to medical devices like insulin pumps and pacemakers, to computer peripherals like printers, scanners and routers, to communication equipment like radios and cellphones, to household appliances like television sets and refrigerators, to various kinds of vehicles and so on. In the past 15 years, security researchers and/or hackers have remotely broken into systems in each of these categories [1–9]. For example, in 2011 Jerome Radcliff showed he could wirelessly hack into his insulin pump and cause it to deliver incorrect dosages of his medication [3].

As an example of this trend, we discuss in more detail the cybersecurity of automobiles. Modern automobiles are essentially computers on wheels. A typical car manufactured today has somewhere between 30 and 100 embedded control units (ECUs). An ECU is just a computer: some are very small and run specialized software on bare metal, while others are general-purpose processors running desktop operating systems like Linux or Windows [10]. The ECUs on a car are connected via a number of CAN bus networks operating at different speeds. It is tempting to consider the high-speed networks as high-security, but that is inaccurate. When applied to CAN buses, the names ‘high’ and ‘low’ refer simply to transmission speed, not security level.

In 2010, Tadayoshi Kohno and Stefan Savage showed that the CAN buses on an automobile are all connected, allowing every ECU to talk to every other ECU. Furthermore, once connected to the CAN bus network, a hacker can replace the software on any of the connected ECUs [8]. Specifically, they showed that if they could plug into the on-board diagnostics (ODB-II) port, required by law to be under the steering wheel on all cars built for the US market since 1996 [11], they could use ECUs to bridge all the networks in the car and reflash the software on every ECU.

For a variety of compelling reasons, most of the functionality of a modern automobile is controlled by software. For example, brakes are controlled by software to enable anti-lock braking, which has been shown to dramatically improve car safety [12]. Acceleration is controlled by software to enable cruise control. Steering is controlled by software in cars with a self-parking option or advanced lane-following cruise control. Door locks are controlled by software so that car manufacturers can remotely unlock cars for customers who have locked their keys in the car. These worthy features are all made possible by software control. Unfortunately, implementing these features in software means that hackers who succeed in breaking in can take over control of braking, acceleration, etc.

In 2011, Kohno and Savage showed they could achieve the same effect, replacing all the software in a car, *without physically touching the car* in four different ways [9]. First, they could put a virus on a computer used for diagnostic purposes in a repair shop. When mechanics connect the computer to a car via the ODB-II port, the virus spreads to the car and allows the attackers to take control just as if they had a direct physical connection. Second, they could exploit a strcpy bug in the car’s Bluetooth interface provided to support hands-free dialing. Exploiting this bug requires pairing an attacking device to the car, a process that can be brute-forced at the rate of eight to nine PINs per minute. Third, they could break in through the telematics unit. Telematics is the generic term used in the automotive industry to describe a service that provides continuous connectivity between cars and a monitoring station. Such services (e.g. BMW’s BMW assist, Ford’s sync, Lexus’ Enform, GM’s OnStar, Mercedes-Benz’ mbrace, Toyota’s SafetyConnect) offer a variety of useful features such as arranging for assistance in the event of an accident, tracking the vehicle if it is stolen, diagnostic reporting and unlocking the car when the owners lock their keys inside. Cars with telematics units have a cell phone interface to provide remote and on-going network connectivity between the vehicle and the monitoring station. Kohno and Savage demonstrated an approach that requires on average 128 phone calls to the car to break an authentication protocol, after which they can rewrite the software to allow future communication without any authentication. Fourth, they were able to break in through the car’s entertainment system. They created a digital version of a song that played perfectly on a PC but that enabled remote code execution when it played on the particular CD player in the car. The digital encoding of the song contained extra information that triggered a buffer overflow on the car’s CD player.

In their work, Kohno and Savage do not disclose the make or model of the car they attacked, in large part because they felt the vulnerabilities were not particular to the car they studied but rather common across the industry. Subsequent reports by other researchers have supported their initial assessment [13–16]. In 2015, for example, Charlie Miller and Chris Valasek exploited vulnerabilities in the telematics unit of a Jeep Cherokee, taking remote control of the vehicle while a reporter from *Wired* magazine was driving the car on a highway to meet them [17]. During the drive, the hackers adjusted the air conditioning, the radio and the windshield wipers. Eventually, they disabled the transmission, which meant the driver could no longer control the speed of the car, which slowed to a crawl and caused a potentially dangerous situation. This demonstration did not show any fundamentally new security issues with automobiles. It did, however, lead to the recall of 1.4 million vehicles.

## 2. Computer security is hard

Researchers and hackers can electronically break into cars in particular and computers in general because it is hard to build computer-based systems with good security. The difficulty comes from the complexity, flexibility and connectivity of computer systems. System designers have to do many things well at many different levels to produce a system with good security. They have to worry about everything: the architecture of the system, how it is specified, how it is coded, how it is configured, whether its users can be trusted not to misuse the system either ignorantly or maliciously, the strength of the passwords used to access the system, the existence of side channels, how the system is physically protected, the security of third-party software and how it interfaces with the rest of the system, the security of the underlying hardware, etc.

In this paper, we focus only on security problems arising from implementation errors. Although only one aspect of security, it is an important one because bugs in software can be converted into exploits that attackers can use. For example, the IBM X-Force reports that of the approximately 8000 reported vulnerabilities in 2012, more than 42% had public exploits available [18]. (Some vocabulary: a vulnerability is a flaw in a computer system; an exploit is an attack that converts one or more vulnerabilities into something that allows attackers unauthorized access to a system or information it contains.)

Clever hackers can convert implementation errors into exploits that can execute arbitrary code on victims’ computers or exfiltrate information. For example, Microsoft released a security bulletin in 2015 announcing that a buffer underflow bug in the Adobe Type Manager Library allowed attackers to remotely install and run code. The vulnerability affected every single Windows platform that Microsoft supported at the time [19]. In 2014, security researchers at Codenomicon and Google independently found Heartbleed, which is a vulnerability arising from a missing bounds check in OpenSSL [20]. This vulnerability allows attackers to exfiltrate secret keys and other sensitive information from any computer running a widely used cryptographic software library. The attack leaves no footprint, so users cannot audit their systems to determine if they have been attacked. MITRE’s common vulnerabilities and exposures (CVE) list gives many more examples of similar exploits [21].

Converting vulnerabilities into exploits is not easy: it requires very specialized skills that most people do not have. However, entrepreneurial black-hat hackers have created black markets for ‘Exploit Kits.’ These kits enable less skilled attackers to break into computer systems without having to discover their own exploits. For example, the Phoenix Exploit Kit from 2010 combined 10 different CVE vulnerabilities into a tool for exploiting systems [22]. The emergence of such kits raises the threat caused by security vulnerabilities because the kits allow people with limited technical skills to launch cyber-attacks.

## 3. Hypothesis: formal methods can help

For decades, formal methods have offered the promise of software that does not have exploitable bugs. For decades, the techniques have not been able to deliver on that promise. In this section, we describe four reasons why now is a good time to revisit this hypothesis. The first is the exponential growth in the numbers of transistors and clock speed that computer architects delivered from 1970 until 2000, fulfilling the promise of Moore’s Law. Formal methods often require searching very large spaces, and so larger memories and faster processors make a big difference.

A second reason is increased automation, much of which has its roots in the seemingly simple problem of Boolean satisfiability, usually called SAT. This problem asks whether it is possible to find an assignment of truth values to Boolean variables that makes a Boolean formula true. This problem is relevant to formal methods because many important questions about software and computer systems can be encoded in Boolean formulae. The formulae tend to be very long; a standard technique is to encode each bit in a relevant portion of memory as a Boolean variable. To the extent that an algorithm can find satisfying assignments, properties about the software can be proven automatically. In general, the SAT problem is NP-Complete, meaning the only known solution is to enumerate every possible truth assignment. In practice, however, many instances of this problem can be solved efficiently using heuristics. This insight has led to an annual competition in which researchers from around the world vie to show their SAT solver is the best. Competition organizers create a corpus of challenging Boolean formulae; the entry that solves the most problems in 20 minutes wins the competition.

**Figure 1.**
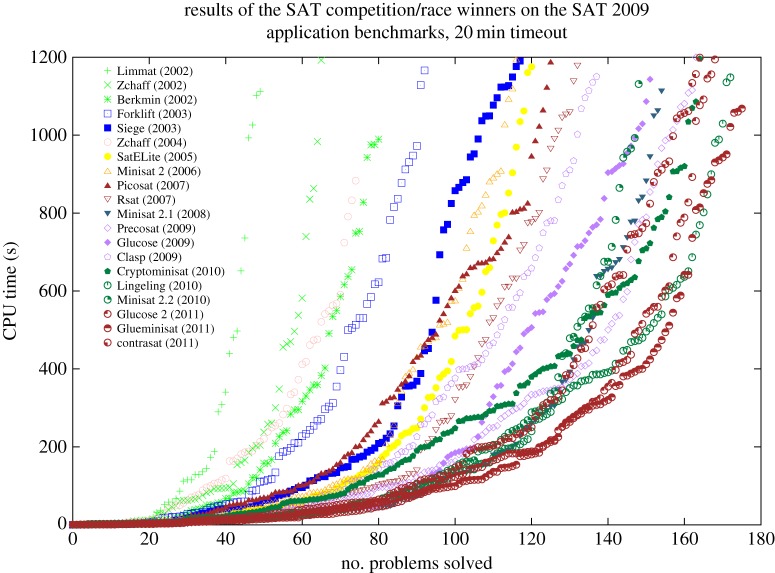
Performance improvements of SAT solvers 2002–2011. Points further to the right correspond to better solvers. Figure taken with permission from Le Berre and colleague’s report on SAT competitions [23]. (Online version in colour.)

In 2011, Daniel Le Berre and colleagues conducted an experiment that reveals just how much SAT solvers improved in a decade’s time [23]. They took the winning SAT solvers from 2002 through 2011 and ported them to a 2011-era machine. They then ran these 20 solvers on the competition problems from 2009, producing the graph shown in figure 1. The horizontal axis represents the number of problems solved; the vertical the time required to have solved the corresponding number of problems. Each point (*n*,*t*) for a solver *s* represents the cumulative time *t* that *s* required to solve *n* problems, so points further to the right represent better solvers. To see the magnitude of the improvement, consider the 80-problem point. The best solver in 2002 took approximately 1000 s to solve 80 problems, while the best solver in 2011 took only 40 s, an improvement of two orders of magnitude.

SAT solvers form only the most basic level of automation in formal-methods tools. SMT (Satisfiability Modulo Theories) solvers [24] add the ability to automatically reason about higher level structures such as integers, vectors and arrays. Tactic libraries allow interactive theorem provers to automate parts of the proof construction process [25].

A third reason that now is a good time to explore using formal methods is that the infrastructure has become much richer. In the past, formal-methods researchers had to invent tools *and* use them to prove properties about software of interest. Since the tools themselves were nascent research projects, the capacity to prove interesting properties was lacking. Now, the situation has improved: many robust formal-methods tools are publicly available with suitable documentation and licences. Examples include ACL2, Alt-Ergo, Astree, Bedrock, Boogie, Coq, CVC4, Frama-C, Isabell, KLEE, PVS, SLAM, TLA+, VCC, Yices2, Z3 and many others. These tools are sufficiently developed that they can be productively used by people other than the original developers.

A fourth reason is more subjective. Critical systems are reaching a level of complexity that makes it apparent that more advanced tool support is necessary. For example, system developers at Amazon Web Services started using model checking to reason about their distributed systems:

We have found that testing the code is inadequate as a method to find subtle errors in design, as the number of reachable states of the code is astronomical. So we looked for a better approach [26].

They point out that human intuition is poorly suited to reasoning about extremely rare ‘once in a million’ kinds of events, but such events happen every second in systems as large as Amazon Web Services. Formal methods help find the strange corner cases and allow designers to decide what to do in those cases.

## 4. Some evidence: DARPA’s high-assurance cyber-military systems program

The premise of DARPA’s high-assurance cyber-military systems (HACMS) program was that systems built using formal methods could be significantly more secure than current norms. Such systems would not have many of the kinds of bugs that hackers currently exploit: buffer over- and under-flows, integer over- and under-flows, etc.

The threat model used in HACMS was that attackers do not have physical access to the system, but they do have complete knowledge of it, including the design process, the architecture and all source code. The program focused on remote attacks because those attacks have the potential to scale: many systems could be attacked simultaneously across a wide geographical area. Physical attacks of comparable scale necessarily require more resources on the part of the attacker. For purposes of limiting the scope of the program, hardware was assumed to be correct.

HACMS researchers focused on pairs of platforms: one open-source ‘experimental’ vehicle and one proprietary or otherwise restricted ‘transition’ vehicle. All researchers on the program were allowed complete knowledge of and access to the experimental platforms, while only researchers with organizational connections to the transition vehicles could access them. This structure created a workflow in which researchers developed tools and techniques and demonstrated them on the experimental platforms. Transition vehicle experts would then apply the tools and techniques to the proprietary platforms. For example, the HACMS ‘Air Team’ used as their experimental platform an open-source quadcopter and as their transition platform Boeing’s Unmanned Little Bird (ULB), which is a helicopter large enough to accommodate up to two (optional) pilots.

The HACMS program commenced with professional penetration testing experts, a so-called ‘Red Team,’ trying to break into each of the HACMS vehicles to build a baseline security assessment. The Red Team was given six weeks of unrestricted access to the vehicles, including documentation and source code. At the end of that time, the Red Team demonstrated serious vulnerabilities in all of the platforms. For example, they were able to connect to the quadcopter while it was in flight, instruct it to not listen to its legimate operator, crash the operator’s computer and then manipulate the quadcopter as if they were the legitimate operator. This lack of security in the quadcopter is not surprising. It is likely that the manufacturer was more concerned with ensuring someone could always control the quadcopter rather than preventing unauthorized individuals from taking control.

The challenge faced by the HACMS researchers was how to modify the software in the program vehicles to make it high-assurance. The program was structured as three 18 month phases. At the end of each phase, the researchers had to deliver working versions of each of the program platforms to the Red Team. The Red Team assessed these vehicles for functionality (Did the system still do what it was supposed to do?) and for security (How hard was it to trigger vulnerabilities?).

### (a) Phase 1

**Figure 2.**
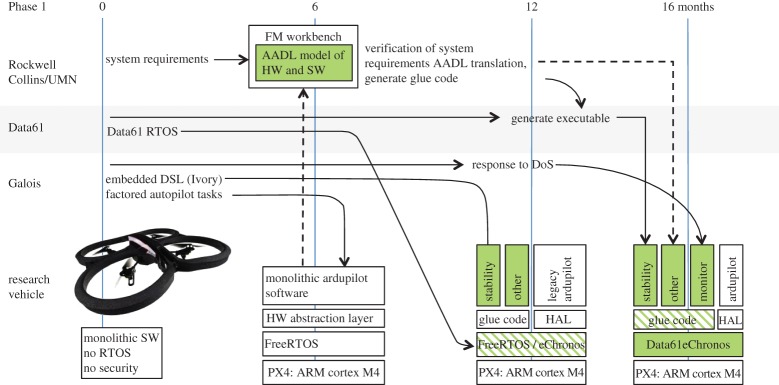
Phase 1 architecture of the SMACCMCopter. Green boxes denote high-assurance components.

In Phase 1, Air Team researchers modified the quadcopter as shown in figure 2, producing the renamed, high-assurance ‘SMACCMCopter’ at the 16 month mark for Red Team evaluation. The Air Team started with a commercially available ArduCopter that had a monolithic software stack, no real-time operating system (RTOS) and no security. Within the first six months, they replaced the underlying Arduino chip with a slightly more powerful PX4:Arm Cortex M4. They refactored the software to use FreeRTOS, an open-source RTOS, and defined a hardware abstraction layer (HAL) so the remaining autopilot software didn’t need to know if it was running on the original or reconfigured hardware. Next, they started replacing legacy software with high assurance versions. Data61 (then called NICTA) designed and built a high-assurance real-time operating system called eChronos [27]. Galois created Ivory, a sub-Turing complete domain-specific language (DSL) embedded in Haskell for writing memory-safe C-code [28]. In this new language, they rewrote many aspects of the ArduCopter’s software, including its flight control software and its code for communicating with the ground station. They added a module to detect denial of service attacks manifested as an attacker flooding the communication channels. Galois also created an embedded DSL called Tower that extends Ivory with the ability to describe concurrent tasks, their properties and the connections between them [28]. The Tower compiler generates memory-safe, machine-specific, low-level C code for connecting the SMACCMCopter’s tasks. Tower also generates models of the system’s architecture in the AADL language [29] suitable for high-level reasoning. Rockwell Collins and the University of Minnesota built two formal-methods tools integrated with the AADL environment called Resolute [30] and AGREE [31]. Resolute uses models written in AADL to build assurance cases and organize proofs about system-wide properties. AGREE performs compositional verification of system properties based on assume-guarantee contracts added to the AADL models. A trusted build tool leverages the models provided by Tower to generate additional glue code to connect together all of the SMACCMCopter software.

The SMACCMCopter at the end of Phase 1 had almost but not all of the functionality of the original ArduCopter. Specifically, it had stability control, altitude hold, directional hold and about 80% of GPS waypoint navigation implemented. In addition, the researchers had added the ability to detect a DOS attack and respond in a mission-specific way. In all, the system had approximately 80 000 lines of high-assurance code.

The Air Team proved a number of system-wide security properties. First, they proved that the SMACCMCopter is memory safe, which means that the myriad attacks that leverage the lack of memory safety do not work against the SMACCMCopter. Second, they proved that the SMACCMCopter will drop any non-authenticated or malformed message it receives, raising the security level of the system considerably. Now, for attackers to get the system to respond to their messages, they must have the appropriate credentials and they can only send a relatively small set of messages that the system is expecting to receive. Finally, they proved the liveness property that any authenticated, well-formed message that reaches the quadcopter will eventually be acted upon by the motor controller. (Note the claim does not ensure all messages sent by the ground control station will eventually reach the motor controller. Jamming can prevent messages from reaching the SMACCMCopter.)

At the end of 16 months, the Air Team delivered the SMACCMCopter to the Red Team for analysis. The Red Team had six weeks to try to break into the system. As at the start of the program, they had full access to the source code and all documents related to the design of the system. Hardware attacks and attacks that required physical access to the copter were out of scope. They were charged with finding a way to wirelessly disrupt the operation of the copter. By the end of this six week period, they were not able to hack into the SMACCMCopter. A penetration testing expert at DARPA commented that the SMACCMCopter is probably ‘the most secure UAV on the planet.’

All code for the SMACCMCopter is available on the web with an open source licence [32].

### (b) Phase 2

During Phase 2, the Air Team updated the architecture of the SMACCMCopter in two distinct ways to better reflect the structure of the Boeing ULB helicopter. First, the hardware was expanded to two processors connected via a CAN bus. One of the processors became the flight-control computer, managing the stability of the vehicle and flight operations. The other acted as a mission-control computer handling communications with the ground station and directing autonomous flight plans. Second, the mission computer software was augmented to include unverified ‘legacy’ components to mimic the reality of how systems are built. Rarely will designers be able to replace 100% of the software on a system, so verified components must be able to protect themselves from possibly compromised components. Concretely, a Linux-based vision application with access to low-level drivers for camera and WiFi hardware was chosen as a representative unverified component.

To provide verified non-interference between components, the mission computer ran Data61’s formally verified seL4 microkernel [33], configured to provide two separate partitions. The first partition comprised the verified security-critical code, including all code for communicating with the ground station. The second partition was configured to run Linux and code for the vision application. Researchers at Boeing configured the ULB software similarly.

At the end of Phase 2, Air Team and Red Team researchers conducted flight and security tests, not only of the SMACCMCopter but also of the ULB. The ULB flight tests were 18 months earlier than originally planned! In the ULB flight test, the safety pilot detected no difference in the handling of the ULB when running HACMS code, demonstrating that, as hoped, formally verified code can be fully performant in practice. In the security tests, the Red Team started with full knowledge of the system and its source code. In this phase, not only were they tasked with remotely breaking into the vehicles, but they were also asked to conduct a much more stringent security test. Specifically, they were given root access to the Linux partition, which communicated with multiple hardware components. This access enabled the Red Team to insert whatever code they wanted into the Linux partition and have it run with administrator privileges. Clearly they would be able to disrupt the vision application. The question was whether they could do worse. At the end of six weeks, the Red Team reported they were unable to break into the vehicle remotely. More significantly, even with root access to the Linux partition, they were unable to break out of their partition or disrupt the operation of the vehicles in any way. In a particularly dramatic test, the Red Team launched a full-scale cyber-attack from their onboard vantage point on the SMACCMPilot while it was flying. As expected, the unprotected vision application was totally destroyed, but all flight-critical functionality remained unaffected.

The significance of these results is hard to overstate. It has long been clear that security cannot be ‘sprinkled’ on existing systems, but the Phase 2 HACMS techniques promise a path forward in which existing systems can have an engineering *cyber-retrofit*: existing (potentially insecure) components can be reconfigured to operate within a system context that protects them and prevents cascading cyber-security failures. Metaphorically, this approach parallels the methods of earthquake protection, which similarly cannot be accomplished superficially. Instead, in a *seismic retrofit*, buildings can be made earthquake resistant by raising them up, rebuilding their foundations, and then reattaching the original building components to secure infrastructure.

### (c) Phase 3

During Phase 3, HACMS researchers continued to improve the security of their vehicles. In addition, because of the Phase 1 and 2 successes, they began working with a variety of transition partners to get the tools, techniques and exquisite artefacts they have developed incorporated into the development of a number of vehicles and systems of relevance to DARPA. The hope is to create a virtuous cycle, in which new procurement calls from military agencies include requirements for proofs of correctness. Companies wishing to win these contracts have to develop the expertise and tools to deliver this kind of assurance. Once they have this kind of capability, they can re-use it to deliver high-assurance civilian products like insulin pumps and pacemakers.

Improvements to the SMACCMCopter include the completion of a formal model of its architecture, updating seL4 on the mission computer with real-time extensions, using Data61’s eChronos [27] real-time operating system (RTOS) on the flight control computer, and implementing high-assurance geofencing. Geofencing is a monitor to constrain bad behaviour that is the result of either a successful cyber-attack or a latent software error. It overrides other behaviour to prevent the SCMACCMCopter from wandering out of a defined geographical area.

The ULB flight-control computer has had its control-laws’ outer loops replaced with software generated from the Ivory DSL [28]. Also, the ULB has been given high-assurance geofencing as well as a high-assurance determination of when the vehicle is on the ground. This software has been proved to be functionally correct, and it is protected from tampering by the seL4 kernel [33]. These software modules can be relied upon to prevent bad behaviour from malicious or faulty software. The end of Phase 3 demonstration showed how this new functionality in concert with the HACMS-generated improvements to the ULB can thwart attacks originating from malicious insiders or from the vehicle’s supply chain. Specifically, neither a turncoat maintenance person loading malware nor a camera module pre-loaded with malware could cause the ULB to operate outside of its intended zone of operations.

During Phase 3, HACMS researchers undertook several transition studies to demonstrate the broad applicability of the HACMS methods, tools and software. Each transition study worked with a military system program. The depth of each transition study varied, depending upon the target of the study. Every study produced a report detailing which HACMS-developed technologies could benefit the study target. Some studies included a proof-of-concept demonstrating the application of HACMS technologies to some subset of the system. Often the proof-of-concept involved HACMS technology developers working in collaboration with target system subject matter experts. This workflow had the benefit of getting these technologies into the hands of the eventual end users. The targets of these studies included small tactical ground robots, appliqués to military transport vehicles to render them autonomous, the human–machine interface to ship-board supervisory control and data acquisition (SCADA) systems, networked weapon systems and satellites.

## 5. What are formal methods?

Although it may sound like it, formal methods are not a magic wand. According to Formal Methods Europe:

Formal methods are mathematical approaches to software and system development which support the rigorous specification, design and verification of computer systems. [34]

Key aspects of this definition are that formal methods are based on rigorous mathematics and can be used to prove properties of computer systems. The techniques can be applied to both software and hardware. Sometimes they are used directly on the code implementing a system; other times they are applied to higher-level models. The proofs are generally machine-checkable, providing a high degree of confidence that they are right. It is important to realize that *the results only apply if the assumptions are satisfied* and *the guarantees may be weaker than needed*. The assumptions include the extent to which models accurately and completely capture all the relevant aspects of the underlying system.

**Figure 3.**
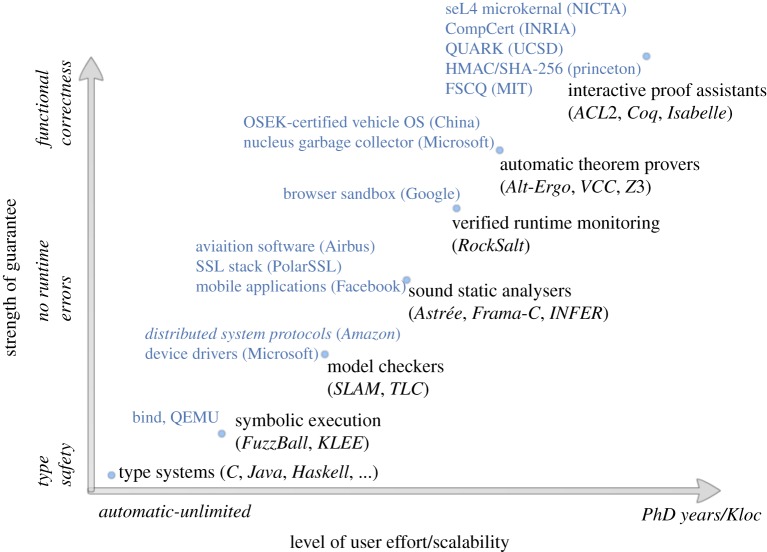
Formal-method tools. Tool classes and example tools are to the right of the plotted points. Example systems analysed using a particular type of tool are on the left. (Online version in colour.)

There are a whole range of different kinds of formal methods, as shown in the notional graph in figure 3, including type systems, model checkers, sound static analysers, verified runtime monitoring, automatic theorem provers and interactive proof assistants. The horizontal axis of the graph shows how much effort is required to use a particular tool, with automatic techniques that can scale to as much code as we can write on the left and labour-intensive tools that require PhD-level expertise and currently scale to programs on the order of 100K lines on the right. The vertical axis shows the strength of the guarantees, ranging from simple type safety properties at the bottom to full functional correctness at the top. Not surprisingly, the most scalable techniques (type systems) provide the weakest guarantees, and the most labour-intensive techniques provide the strongest (interactive proof assistants).

## 6. What software is worth verifying?

Verifying all software to the level of full functional correctness is well beyond current capabilities. More, it seems unlikely that we would ever be able to verify all software to such a level. However, most software does not need to be verified. The right question is *What software is worth verifying*? The answer is that the most important artefacts to verify are those we can leverage to build systems we can trust even in the face of attackers. Appropriate candidates include separation kernels, hypervisors, real-time operating systems (RTOSes), compilers, file systems, web broswers, sandboxes, cryptographic algorithms and garbage collectors. Perhaps surprisingly, the literature already includes artefacts that have been verified in all of these categories, albeit with somewhat limited functionality: the seL4 microkernel [33], the mCertiKOS hypervisor [35], the eChronos and ORIENTIAS RTOSes [27,36], the CompCert C Compiler [37], the FSCQ and BilbyFS file systems [38,39], the Quark web broswer [40], the RockSalt browser sandbox [41], various crytographic algorithms [42,43] and the Nucleus garbage collector [44]. The existence of two of these artefacts were crucial to DARPA’s decision to fund the HACMS program, serving as a basis of confidence that the program had some chance of succeeding: the seL4 microkernel and the CompCert verifying C compiler. We briefly discuss each of them in turn.

### (a) seL4 [33]

A microkernel is the core of an operating system: the part that absolutely has to run in privileged mode. The seL4 [33] microkernel provides virtual address spaces, threads, inter-process communication (IPC), a memory management model and capabilities to manage authorizations. A team of operating systems and formal-methods experts at Data61 (previously NICTA) collaborated on its design, implementation and proof of functional correctness. The microkernel is implemented in about 10K lines of C code. The proof of correctness required approximately 480K lines in the Isabelle/HOL interactive proof assistant. The seL4 microkernel is available with an open-source licence [45]. The team estimates that designing and implementing the microkernel took two person years. Proving it correct required 11 more. They spent an additional nine person years researching and improving the formal-methods infrastructure they used. They paid particular attention to the IPC mechanism because its speed is the crucial performance metric for microkernels. They eventually added and proved correct an IPC fastpath, enabling them to achieve 227 cycles for the standard one-way IPC measurement benchmark, compared with 206 cycles for the unverified OKLA 2.1 microkernel. In addition to proving functional correctness for seL4 extended with an IPC fastpath, the team proved the system correctly enforces access controls, providing integrity and authority confinement. They also proved a form of information-flow non-interference, showing that seL4 can be configured as a separation kernel. HACMS researchers leveraged this capability during Phase 2, allowing the Red Team root access in one partition knowing that the separation properties of seL4 would prevent them from breaking out. Finally, the seL4 team showed that the binary generated from the C code preserved the source semantics, removing the compiler from the trusted code base.

### (b) CompCert [46]

The CompCert verifying C compiler is a ‘lightly-optimizing’ compiler for the subset of C used by the aviation industry. This subset is quite large; the most significant omissions are support for concurrency and separate compilation, both of which are current research topics. Xavier Leroy at INRIA implemented CompCert and proved it correct in the Coq proof assistant, extracting the compiler from the correctness proof using a feature of Coq. The implementation and correctness proof together total 42K lines of code and took three person years to build. CompCert is available online for evaluation and can be purchased for commercial use [37]. Code produced by Compcert is roughly twice as fast as code compiled using gcc at optimization level zero. Compcert-produced code is 7% slower than code produced with gcc -O1 and 12% slower than gcc -O2. Work is underway to enable the use of CompCert in the aviation industry [47], which requires qualification according to standards outlined by DO-178.

## 7. Impediments

As just discussed, recent efforts have shown that formal methods can be used to prove functional correctness for practical systems, yielding software with better safety and security properties. However, significant impediments to using formal methods remain, even for safety- and security-critical software. In this section, we discuss some of those impediments.

### (a) Expertise

One challenge is the lack of people trained in using formal methods. Many formal-methods techniques remain the purview of researchers with PhDs. A study conducted in 2006 estimated there were fewer than 5000 experts in formal methods in the entire world [48]. That situation may be changing, with a number of universities starting to offer undergraduate courses in interactive proof assistants. The recently published electronic book Software Foundations [49], co-authored by people currently at 14 different institutions, provides an interactive, tutorial introduction to theorem proving in Coq. Other guides on using theorem provers have appeared recently [50,51].

### (b) Level of effort

Another impediment to adoption is the level of effort required to complete a correctness proof. Table 1 shows code and proof sizes as well as reported development times for eight formally verified systems. The data show that verification introduces significant extra effort in the size of the project, measured in proof size, which is necessarily reflected in increased development times. Interestingly, seL4 is something of an outlier, with other systems being verified at lower overhead rates. That said, seL4 is a practical system being adopted in industry while the rest of the entries, with the exception of CompCert, are research prototypes. The higher overhead could be a reflection of the robustness of the software rather than of the relative difficulty of verifying a microkernel.

**Table 1.** Required level of effort to verify various artefacts and performance characterization. Data taken from cited papers. The table is sorted by the combined size of code and proof. LoC, lines of code; LoP, lines of proof; LoC+P, the combination used when the source paper did not break out the two; PY, person years.

artefact	size	dev. time	performance
seL4 [33]	10K LoC, 480K LoP	13 PY	206 versus 227 cycles
CompCert [46]	42K LoC+P	3 PY	2× speed of gcc -O0
			7% slower than gcc -O1
			12% slower than gcc -O2
FSCQ File System [38]	24K LoC+P	<5 PY	80% of xv6 file system
certiKOS Hypervisor [35]	2K LoC, 18.5K LoP	1 PY	<2× slowdown on most
			lmbench benchmarks
SHA-256/HMAC [43]	407 LoC, 14.4K LoP	n.a.	equivalent to OpenSSL 0.9.1c
Rocksalt Sandbox [41]	100 LoC, 10K LoP	<2 PY	1M instructions per second
			faster than Google’s checker
Nucleus GC [44]	6K LoC+P	0.75 PY	‘competitive’
Quark Web Browser [40]	5.5K LoC+P	0.5 PY	24% overhead w.r.t. WebKit
			baseline on 10 Alexa Websites

It is interesting to speculate as to why seL4 required so much more effort than verifying CompCert as they are both production quality. At least two factors contribute. First is the ease of specifying correct behaviour. Formally writing down what it means for a microkernel to be correct cannot be done concisely. Try it! By contrast, compiler correctness can be concisely described as requiring the semantics of the generated code to be a refinement of the semantics of the source. This difference means specifications for seL4 are lengthier than the specifications for CompCert. Second, by their very nature, microkernels are not modular, which complicates both their implementation and their proof of correctness. By contrast, compilers are very modular: as typical for a compiler, CompCert is structured as a series of more than 10 independent passes over a representation of the program. This structure means the proof of correctness can similar be structured as 10 independent proofs.

Speculation aside, the right question to ask is whether the increased effort is a good investment. This question hinges not only on the development cost but also on the cost of flaws in software. Calculating the cost of such flaws is not easy! The safety-critical software industry has some understanding of the costs of safety failures based on random, independent failures as the threat model. Such software has traditionally been subjected to careful, process-based certification requirements (e.g. DO-178), which dramatically increase development costs. *Some* of this effort could be redirected to verification, possibly improving quality and potentially even reducing cost. By contrast, the security cost of flawed software is much more difficult to measure. In this case, the threat model needs to be of an intelligent and informed adversary who can drive a system into its failure modes. Another factor is that traditionally society has not held software authors responsible for flaws in their code. If software companies in certain industries were responsible for damages resulting from buggy code, the costs of verification might become a very good investment.

### (c) Performance

Another barrier to the adoption of formal methods is the concern that the performance of the verified system may not be competitive. The final column in table 1 gives a performance assessment of each of the verified systems. Typically, the verified system is somewhat slower than the non-verified version, although not always, cf. SHA-256/HMAC and Rocksalt. Sometimes a formally verified system can leverage a proved invariant to remove unnecessary runtime checks that would otherwise slow performance. In general, however, verified software is slower. It is worth considering why that might be. Generally, developers study performance-critical code carefully, and they write fastpaths to expedite important cases. Each such special case introduces more code that must be verified, with a corresponding increase in the required proof effort. Consequently, formal-methods researchers have introduced only those fastpaths necessary to get ‘good enough’ performance, stopping before achieving parity. In other words, verified code is not intrinsically slower, but verifying faster code can be more time consuming. It is also worth noting that it is impossible to achieve an ‘apples to apples’ performance comparison between verified and unverified systems, in that we cannot be sure that the unverified system is behaving correctly. At the limit, the unverified system could be doing the wrong thing very quickly.

### (d) Time to market

A fourth concern is the delay introduced by using formal methods, corresponding to the additional time required for verification on top of the usual development life cycle. Many of the systems listed in table 1 lack features, which can be seen as another form of delay. In many industries, the time to market is a critical competitive consideration (although likely less so in safety- and security-critical sectors). Unless required to do so by regulation, liability considerations, or insurance requirements, proving full functional correctness is likely to always be prohibitively expensive for some sectors, for example web-based entertainment.

There is a continuum of formal-method techniques, however. Tools requiring lower levels of effort can be useful to a much broader audience. For example, Facebook has built and deployed INFER [52], which is a sound static analyser. INFER can process millions of lines of code and thousands of diffs per day. It requires 4 h to analyse the complete Facebook Android/iOS code base. More importantly, it takes less than 10 mins to process a single diff, which allows the tool to be integrated into the standard Facebook development process. When developers try to check in modifications, INFER runs and the developers are required to address any issues INFER finds *before* they can complete their check-in, which ensures that certain kinds of bugs never enter the production code base. In exchange for this speed, the properties that INFER proves are relatively weak: only the absence of null pointer exceptions and resource leaks.

### (e) Violated assumptions

A fifth challenge lies with the assumptions in proofs. If the assumptions are wrong or not satisfied by a particular system, then the verification results do not apply. Unreasonable assumptions (or too-weak guarantees) can make formal results useless. John Regehr is a programming language researcher who has developed the Csmith tool that uses randomized techniques to stress-test compilers. In 2011, he reported finding 325 bugs in various C compilers, *including CompCert* [53]! The bugs in CompCert were in the unverified front end, which has subsequently been fixed and verified [37], and in the hardware model. Both of these bugs correspond to assumptions in the original functional correctness proof for CompCert. Despite these flaws, the CompCert compiler performed very well in Regehr’s tests:

The striking thing about our CompCert results is that the middle-end bugs we found in all other compilers were absent. … CompCert is the only compiler we have tested for which Csmith cannot find wrong-code errors. This is not for lack of trying: we have devoted about six CPU-years to the task. [53]

## 8. Taking stock

### (a) Lessons learned

In reviewing the verification projects listed in table 1, certain themes emerge that may eventually become best practices.

*Don’t verify existing code artefacts*. Verifying existing artefacts is more difficult than co-developing a system and its correctness proof. Two factors contribute to this phenomenon. First, when reasoning about an existing system, verification experts have to discover the required properties by spelunking through the code base and documentation. When reasoning about a system being co-developed, the required properties are (relatively) accessible. Second, when coding a system, developers face many choices in how to do things. When they know they have to produce a correctness proof concomitantly, they can make choices that facilitate verification.
*Don’t verify all code*. Many systems can be partitioned into code whose correctness is essential for overall system security and code whose correctness is less crucial. For example, the HACMS Air Team was able to leverage the security properties of the seL4 microkernel to sandbox Red Team code. The resulting architecture meant that by verifying only a portion of the software on the vehicle, the Air Team could ensure that buggy or malicious third-party software in other portions could not disrupt the operation of the vehicle.
*Use testing to eliminate obvious bugs before attempting verification*. Systems that have bugs cannot be functionally correct, so low-cost tools for finding bugs should be used before starting formal verification. Formal-methods tools are useful for forcing developers to consider all the unusual corner cases where bugs might lurk. This process is more efficient when the obvious bugs have already been eliminated.
*Leverage automation*. Automation, whether it be decision procedures like SAT and SMT solvers or tactic libraries, allows the computer to do part of the verification task, leaving less of the problem to verification experts. Automation also allows proofs to be replayed when parts of the system change, automatically finding places where the proofs need to be updated. Expanding the scope of automated decision procedures and creating more powerful tactic libraries are on-going research challenges.
*Use Domain-Specific Languages (DSLs) to co-generate code and proofs*. Another strategy that multiple verification groups have been using is to write code in DSLs instead of general purpose languages [28,39,41]. DSLs can be designed with limited power to facilitate verification. They can also simultaneously generate executable code and proof scripts.

### (b) On-going research and challenges

The successes described so far suggest that formal methods will eventually allow the construction of practical systems with much better security properties. In the remainder of this section, we briefly describe some of the many remaining challenges.

*Developing and validating models of real-world systems*. Examples include the x86 instruction set architecture, LLVM, POSIX interfaces, various Linux interfaces, browser APIs, etc. Flaws in these models can invalidate proofs of functional correctness, as Regehr’s analysis of CompCert demonstrated. Building these models is labour intensive, but the benefits can be shared by many different applications. Peter Sewell’s group at Cambridge University is a leading force in this area.
*Increasing the level of automation*. Areas of current research with this goal include better SAT solvers, richer theories for SMT solvers and improved tactic libraries.
*Scaling*. Key challenges are developing techniques to scale proofs to larger systems and to manage the large proofs that result. Together these challenges form the nascent field of proof engineering. The researchers at Data61 who developed seL4 and who now must maintain the code and its correctness proofs are taking the lead in creating this new field.
*Integration into the normal development process*. Tools that are part of the standard developer tool chain get used all the time, and so have the opportunity to improve the quality of lots of code, like what Facebook is doing with the INFER project.
*Getting developer buy-in*. When a developer at Amazon Web Services realized the potential of model checking for finding design errors triggered by one-in-a-million-events, he wanted others to adopt the same techniques. He ran a tutorial for his colleagues explaining the approach, but instead of calling it formal methods or model checking, he called it ‘exhaustively testable pseudo-code.’ This neutral name allowed the people in the audience to learn about the technique with an open mind and contributed to its adoption by the larger group [26].
*Concurrency*. Many formal methods work by exhaustively searching a space of possible executions. This approach is computationally very expensive even with single-threaded code, but concurrency leads to a combinatorial explosion in possible states. Different proof techniques are needed to handle this case. A potential saving grace is that many safety- and security-critical can be restricted to single-threaded executions or executions with very controlled concurrency.

## 9. Conclusion

A vision for the future of software construction is that formal methods are used to produce exquisite software artefacts that come with proofs of functional correctness and that can be used to build practical systems with strong safety and security guarantees. The seL4 microkernel and the CompCert verifying C compiler are initial examples of such exquisite artefacts. The SMACCMCopter and Boeing’s upgraded ULB are examples of formal-methods based, practical systems with enhanced security, as measured by Red Team assessments. A hope is that the SMACCMCopter and the ULB will serve as proof that such systems are possible and spur investment in the area, leading to a world in which networked devices are not nearly as vulnerable to attackers as they are at present. Much work remains, but there is hope.

## Data accessibility

This article has no additional data.
